# De novo assembly of transcriptomes and differential gene expression analysis using short-read data from emerging model organisms – a brief guide

**DOI:** 10.1186/s12983-024-00538-y

**Published:** 2024-06-20

**Authors:** Daniel J. Jackson, Nicolas Cerveau, Nico Posnien

**Affiliations:** 1 University of Göttingen, Department of Geobiology, Goldschmidtstr.3, Göttingen, 37077 Germany; 2 University of Göttingen, Department of Developmental Biology, GZMB, Justus-Von-Liebig-Weg 11, Göttingen, 37077 Germany

**Keywords:** Transcriptome assembly, De novo assembly, RNA-seq, Short reads, Emerging model system, Genome, Annotation, Differential gene expression

## Abstract

Many questions in biology benefit greatly from the use of a variety of model systems. High-throughput sequencing methods have been a triumph in the democratization of diverse model systems. They allow for the economical sequencing of an entire genome or transcriptome of interest, and with technical variations can even provide insight into genome organization and the expression and regulation of genes. The analysis and biological interpretation of such large datasets can present significant challenges that depend on the ‘scientific status’ of the model system. While high-quality genome and transcriptome references are readily available for well-established model systems, the establishment of such references for an emerging model system often requires extensive resources such as finances, expertise and computation capabilities. The de novo assembly of a transcriptome represents an excellent entry point for genetic and molecular studies in emerging model systems as it can efficiently assess gene content while also serving as a reference for differential gene expression studies. However, the process of de novo transcriptome assembly is non-trivial, and as a rule must be empirically optimized for every dataset. For the researcher working with an emerging model system, and with little to no experience with assembling and quantifying short-read data from the Illumina platform, these processes can be daunting. In this guide we outline the major challenges faced when establishing a reference transcriptome de novo and we provide advice on how to approach such an endeavor. We describe the major experimental and bioinformatic steps, provide some broad recommendations and cautions for the newcomer to de novo transcriptome assembly and differential gene expression analyses. Moreover, we provide an initial selection of tools that can assist in the journey from raw short-read data to assembled transcriptome and lists of differentially expressed genes.

## Introduction

**Fig. 1:**
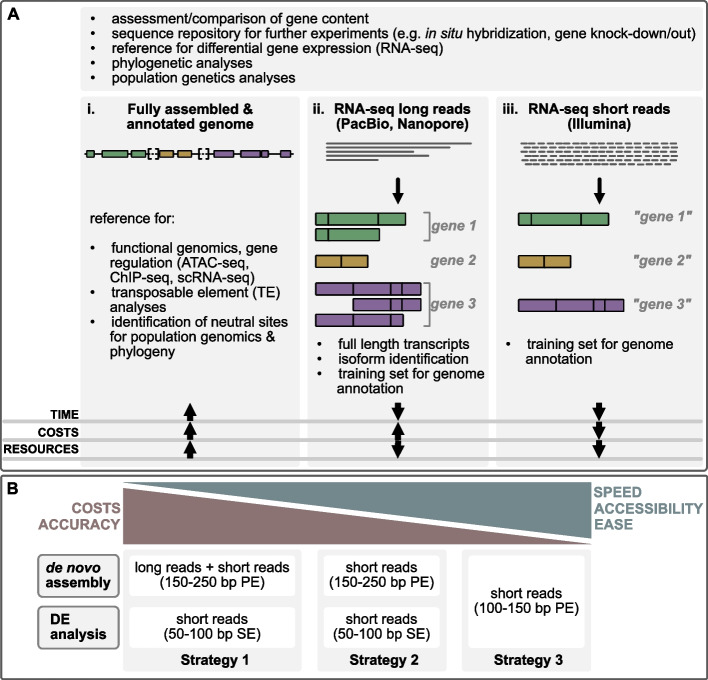
Overview of applications for genome and transcriptome resources. **A** Overview of major applications facilitated by “omics” data. The upper box summarizes applications provided by all methods outlined in Ai-iii. (Ai) Chromosome-level genome with high-quality gene annotation. (Aii) Transcriptome assembly based on long reads (e.g. PacBio Iso-Seq). (Aiii) Transcriptome assembly based on short reads (Illumina, 100–250 bp). **B** Recommendations for experimental design decisions

A major goal in Biology is to understand the processes that underlie the phenotypic variation observed in nature. Because information about organismal phenotypes, such as appearance and function, are stored in the genome, holistic approaches have greatly benefitted from advances in sequencing technologies which provide the opportunity to meaningfully integrate molecular, genetic and morphological data. Accordingly, genomic databases have grown rapidly since the completion of the first animal genome in 1998 [1]. These genomic resources have revolutionized multiple biological disciplines and have generated unprecedented insights into disparate phenomena. For instance, the reconstruction of phylogenetic relationships employing genomic information (i.e. phylogenomics) has resolved previously undetermined relationships and radically changed our view of the tree of life [2]. Cost-efficient sequencing of organismal communities directly from environmental samples (i.e. metagenomics) have provided novel opportunities for the description and monitoring of biodiversity (e.g. [3]), and the analyses of population dynamics, patterns of adaptation and the demographic history of organisms profit from extensive genome re-sequencing (e.g. [4]) (Fig. 1Ai). As a genome contains the information for all protein coding genes, as well as non-coding regulatory sequences (Fig. 1Ai), studies focused on chromatin accessibility (e.g. ATAC-seq, [5]), transcription factor binding sites, histone modifications (e.g. ChIP-seq, [6]) and epigenetic DNA modifications (e.g. bisulfite sequencing for methylation studies, [7]) require a well-assembled genome sequence. Moreover, studies assessing mobile element insertions (i.e. transposable elements; [8]) and the identification of neutral sites for population genomics inferences (e.g. [9]) often require reference genomes.

Despite the exciting opportunities that genome-scale approaches provide, the generation of a complete genome sequence requires considerable financial and computational resources, as well as different sets of expertise to assemble, annotate and analyze the final dataset. Genome assemblies may be challenged by large genome size [10] and high content of repetitive sequences (for example typical for molluscan genomes; [11]). Accordingly, genome assembly approaches often rely on the integration of Illumina short reads and long-read data for the generation of continuous fragments (contigs), which are combined into larger fragments (i.e. scaffolds), preferentially entire chromosomes, by optical mapping approaches or genome wide three-dimensional genomic contact information (i.e. HiC) [12–16]. The success of a genome annotation effort can also strongly depend on the structure of the genome, such as the ratio of coding to non-coding sequences and the availability of gene expression data to train gene model prediction algorithms [17]. Therefore, assembling and annotating a genome de novo is often not achievable for an individual research group, but requires input from different labs usually in larger consortia. Moreover, despite ever-growing genomic databases it remains challenging to establish causative links between a genome sequence and the organismal phenotype it produces [18]. This is in part because the sequence information stored in the genome is transferred to other molecules (e.g. proteins or regulatory RNAs) which only then affect cell and organ morphology and function. For example, the ultimate action of a functional protein is achieved and regulated by multiple molecular processes, such as transcription, translation and post-translational modifications [19]. While sequencing-based methods to study and quantify these regulatory steps have been established in recent years [20], many such functional genomic approaches remain restricted to well-established model organisms. However, as many basic aspects can be well analyzed without a full genomic sequence, high-throughput sequencing of transcribed genes (i.e. transcriptome sequencing) by RNA-seq is broadly used to assess the content and abundance of transcripts in any organism, tissue and more recently in individual cells [21–24] (Fig. 1Aii—iii). Differential gene expression (DGE) studies based on RNA-seq data can be used to quantify differences in transcript abundance across multiple natural (e.g. between species, populations or between developmental stages or habitats) and experimental (e.g. between treatments or genetic modifications) conditions. Therefore, RNA-seq has become a standard approach in all domains of biology to better understand how genetic information defines organismal phenotypes (e.g. [25, 26]).

**Fig. 2:**
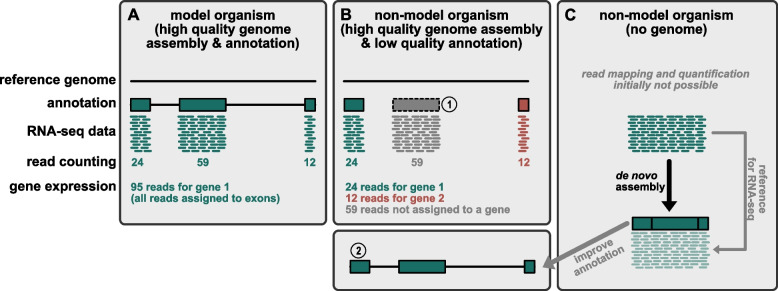
Overview of current challenges during RNA-seq analysis. **A** RNA-seq analysis in a model organism with a high-quality genome annotation. **B** RNA-seq analysis of a non-model organism with a genome reference, but incomplete annotation. 1 – After mapping RNA-seq reads to the reference genome, the additional exon can be annotated, while no information about the connection to the other two exons is available. 2 – Mapping the transcript obtained by de novo assembly of the RNA-seq data onto the genome allows annotating the full gene model. **C** If no genome reference is available, RNA-seq data cannot be readily used to quantify gene expression. A de novo assembly of the RNA-seq is required to reconstruct transcripts, which can serve as mapping references for RNA-seq data and to improve existing genome annotations

Gene expression studies based on RNA-seq are easily performed for model organisms with a reference genome and a high-quality annotation (Fig. 2A). The annotation of a well assembled genome is often based on an initial round of automated annotation, followed by iterative rounds of manual curation by a dedicated community [27–30]. Despite the efforts of large consortia, such as the Darwin Tree of Life [31] and the European Reference Genome Atlas (ERGA) projects [32], to establish genomic resources for non-model systems, the annotation of these genomes often does not reach the high quality of model organism genomes. Accordingly, DGE analyses derived from poorly annotated genomes in these non-model systems may not be the ideal approach; an incorrect or incomplete genome annotation has a major impact on the analysis of gene expression data (Fig. 2B) ([33], and unpublished observation). Moreover, for many emerging model organisms a well assembled and annotated reference genome often does not exist (Fig. 2C). As RNA-seq captures the transcribed regions of the genome, such as protein coding genes and regulatory RNA molecules [21, 34], the establishment of de novo transcriptome resources (i.e. a de novo transcriptome assembly) has become a powerful and time and resource efficient tool to study the gene content of an organism. Transcriptomic resources have been successfully employed to estimate genetic divergence in population genetic studies [35, 36], to conduct meta-transcriptomic surveys of environmental samples [37, 38] and to reconstruct phylogenetic relationships [39–44]. They can also facilitate the efficient identification and molecular isolation of specific gene sequences for further studies, such as in situ hybridization or loss of function experiments using RNA interference (RNAi) [45] (Fig. 1A). Moreover, de novo transcriptome assemblies can serve as a reference for DGE studies (Fig. 2C), and such resources can also be employed to improve genome annotations (Fig. 2B, 1&2). As many emerging model organisms are studied by small research communities, the economic cost and technical challenges of generating a high-quality genome assembly and annotation are not realistically achievable within the timeframe of a typical project (3–4 years), and therefore many of these methods are restricted to well-established and/or well-funded model systems. Therefore, the de novo assembly of transcriptomes remains a valuable approach to study the genetic and molecular underpinnings of phenotypic traits and to improve genome annotation.

Researchers that wish to add a genetic component to their research questions may be overwhelmed by the many decisions that need to be taken to obtain the best possible data. For instance, ‘which next generation sequencing technology should I choose, short reads or long reads? How many replicates do I need for a DGE study? What assembly software should I use and how do I assess the quality of any output?’. In this article we provide an overview of typical workflows for de novo transcriptome assemblies and subsequent DGE analyses. We also highlight some of the challenges associated with study design, sample preparation, de novo assembly, DGE analysis and evaluating the outputs of these exercises. In general, we refrain from making dogmatic recommendations concerning the use of particular software packages and encourage the user to explore the impact that different approaches and tools have on their own data. To this end, we recommend adopting a systematic and organized approach to exploring any RNA-seq dataset so that objective comparisons can readily be made and interpreted. We also refrain from making any cost comparisons or explicit budget recommendations because the available technologies and their corresponding shortcomings, strengths and ‘cost-per-base’ prices are evolving extremely rapidly. Rather we encourage the researcher to inform themselves of current market prices and to explicitly weigh the technical advantages and disadvantages of each technology according to their research needs. Importantly, we are primarily concerned with emerging model organisms for which limited, or no genome or transcriptome resources exist, and we assume this category includes organisms for which there also exists little functional genetic information, such as spatial gene expression or gene function data.

### Generating and using RNA-seq data

**Fig. 3:**
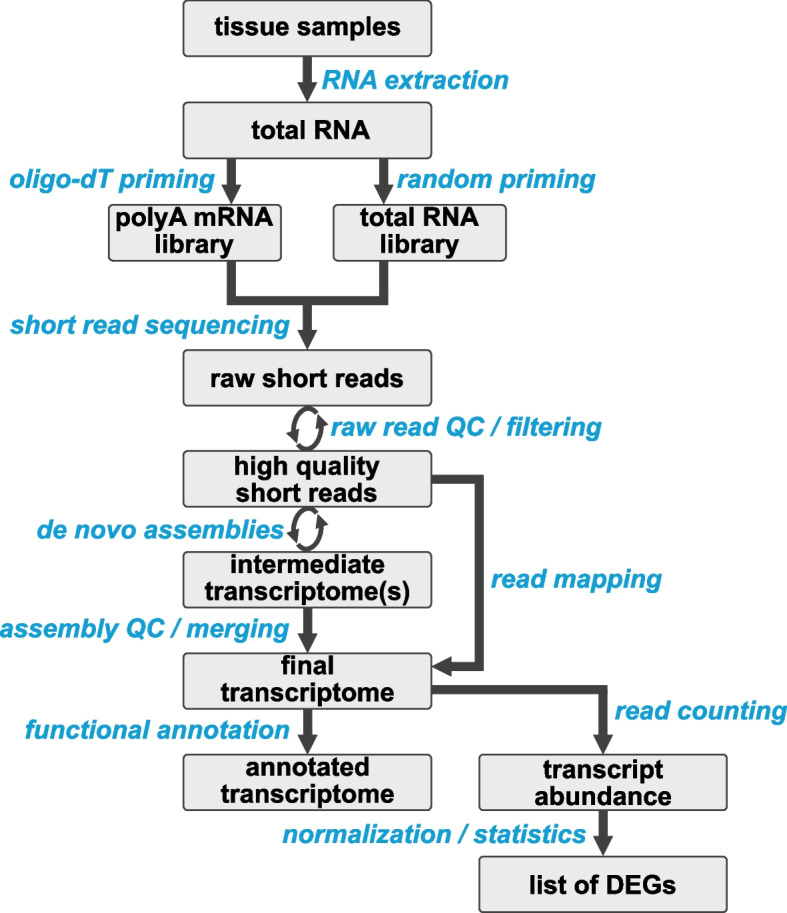
General outline of major steps for de novo assembly and DGE analyses

The generation of RNA-seq data is a cost and time efficient entry point for genetic and molecular analyses in an emerging model system [26]. A typical RNA-seq experiment starts with the isolation of total RNA from the organism, tissue or developmental stage of interest. The RNA molecules are fragmented, reverse transcribed into complementary DNA (cDNA) and sequencing adapters are incorporated. By selecting polyadenylated molecules during library preparation, the fragments can be enriched for messenger RNAs (mRNAs). This depletes many non-coding RNA molecules and the majority of ribosomal RNAs from subsequent analyses. If regulatory RNAs are intended to be studied, it is important to retain the full complement of RNA molecules using random priming during the library preparation (Fig. 3) as many of these molecules are not polyadenylated. The cDNA library is amplified by PCR, and these libraries are then subjected to Illumina short-read sequencing resulting in 50–250 bp single- (SE) or paired-end (PE) reads. The quality of these short reads is assessed, and high-quality reads are assembled to reconstruct the original transcripts (i.e. de novo transcriptome assembly) (Fig. 3). This data can then be used to assess the complement of transcripts expressed in a certain tissue or developmental stage. If RNA extracted from whole bodies or multiple organs at different life stages is used for the de novo assembly, the transcriptome will ideally represent a comprehensive resource for the identification of all transcribed gene sequences, and it may serve as a reference to quantitatively estimate transcript abundance (i.e. DGE) across biological or experimental conditions.

Readers will likely be aware that long-read sequencing technologies, such as PacBio and Oxford Nanopore are an alternative to assembling Illumina-derived short reads [46] to generate a de novo transcriptome. As these long-read technologies sequence the entire transcript from the five-prime untranslated region (5’-UTR) to the polyadenylation tail, they are indispensable when the accurate identification and discrimination of splice variants [47] is the goal (Fig. 1Aii). Time-consuming and computationally intense de novo assembly steps are often not required when working with long reads. However, this data is commonly “polished” with short-read data to improve sequence accuracy. It is also important to note that unfragmented, high-quality RNA is needed for efficient long-read sequencing, which may not always be feasible if field samples or preserved material are all that is available. In addition, estimates of gene expression (whether absolute or relative) are almost exclusively based on high-depth short-read data [48, 49]. This is in part because every transcript should be represented by an individual long read and current long-read technologies generally do not generate this sequence depth to accurately infer global levels of gene expression [50, 51]. Gene expression quantification is further hampered because the proper assignment of long reads to particular isoforms tends to be complicated for emerging model systems without reference genomes [50]. Therefore, current long-read based transcriptomics require higher financial and computational resources than short reads and the choice of data to be generated should thus be aligned with the project goals and funds available (Fig. 1Aii, B).

If funding, computational resources, expertise and fresh material are not limiting, we recommend generating a transcriptomic reference based on a combination of long and short reads from the entire organism and/or multiple tissues and from different life stages to obtain a comprehensive collection of highly accurate, full-length transcripts. Subsequent gene expression analyses should then be derived from short-read data (50 – 100 bp reads, SE mode). If resources are limited, a transcriptomic reference can be generated by the de novo assembly of short reads exclusively. Preferentially, longer short reads (150 – 250 bp) in PE mode are used for the assembly of the reference, and shorter reads (50 – 100 bp) in SE mode are generated for subsequent DGE analyses. The most cost-efficient approach is to use the same short reads for the de novo assembly and for subsequent gene expression analysis. In this case, we recommend generating 100–250 bp reads in PE mode (Fig. 1B). We focus here on typical RNA-seq workflows that aim at generating de novo transcriptome references from short reads which can subsequently serve as reference for differential gene expression (DGE) analyses.

### Generating a transcriptome assembly de novo

#### Planning, sample preparation and short-read sequencing

As gene expression is highly context dependent, the completeness of a transcriptome strongly depends on the biological input material. The planned downstream applications must therefore be carefully considered at this stage of the project. For instance, if the transcriptome should serve as reference for subsequent analyses of embryonic gene expression, the de novo assembly should be based on RNA extracted from preferentially multiple embryonic tissues. If an analysis of total gene content is the goal, for example to support a genome annotation, one should obtain RNA-seq data from as many tissues and life stages as possible. In principle, to generate a de novo transcriptome assembly experimental replication of the tissues/stages is not necessary. If resources are available, we would rather recommend investing in sequencing more samples from different tissues and/or life stages to capture more transcript diversity. However, if the same RNA-seq reads used to assemble a transcriptome de novo will serve for DGE studies, proper biological replication (see below) is a prerequisite. It should also be kept in mind that biological replicates derived from different individuals (especially from a highly heterozygous population) may generate assembly artefacts and significantly increase computing time [52]. If the degree of heterozygosity is high or unknown, we recommend to sequence from as few individuals as possible or try to use individuals derived from inbred lines.

Despite the availability of impressively low-input RNA-seq options, we strongly encourage the isolation of adequate amounts of high-quality total RNA (a minimum of 0.3 µg total RNA at a concentration of 30—100 ng/µl). In our experience there is no advantage for the researcher to prepare the sequencing libraries themselves prior to submitting them to an experienced sequencing center because critical steps can be performed for a better cost and usually to a higher standard by dedicated technicians or pipetting robots. Moreover, most sequencing centers will routinely perform quality control (QC) on all RNA samples submitted by clients and will identify problems associated with RNA extracted from unusual tissues, such as tissues with high enzymatic activity or inhibitors of ligases and/or polymerases. For instance, highly fragmented RNA can be easily identified by assessing the fragment size distribution (e.g. fragment analyzer). It is important to note that many sequencing centers tend to handle RNA extracted from predominantly mammalian or well-established model organisms. Accordingly, a typical first check for RNA integrity that employs estimates of the size and relative abundance of 28 s and 18 s ribosomal RNA (rRNA) fragments [53] ‘fails’ for many emerging model systems as the rRNA fragments tend to be of different sizes in different organisms. If the RNA-seq data will be used in downstream DGE analyses (Figs. 2C, and 3), close consultation with the sequencing center will ensure the sensible pooling of barcoded samples to avoid any flow-cell specific technical biases [54]. In general, a clear line of communication and regular check-ins with the sequencing center are therefore essential to avoid preventable missteps.

Once a high-quality RNA-seq library is prepared, several important decisions concerning sequencing, such as read length, type (i.e. PE vs. SE) and the number of reads (i.e. sequencing depth) need to be made. Generally, longer PE reads (up to 500 bp comprised of 2 × 250 bp PE reads) give more complete and less fragmented de novo assembly results [55]. Deciding on the number of reads to sequence should be informed predominantly by the complexity of the transcriptome (i.e. the diversity of genes expressed) and by the desired sensitivity of the analysis, as greater depth is required to detect rare transcripts. As this information is usually not available for emerging model systems, one can only make educated estimates. A good starting point is to consult RNA-seq experiments performed in close relatives of the species of interest. It may be tempting to follow the “a lot helps a lot” rule, but simulation studies have demonstrated negative effects on the quality of the de novo assembly (i.e. the identification of new transcripts) due to saturation. For instance, in the fruit fly *Drosophila melanogaster* (genome size: ~ 180 Mbp), saturation effects have been observed when about 2 million reads were used for the assembly [55]. Similarly, a de novo transcriptome assembly for the common house spider *Parasteatoda tepidariorum* (genome size: ~ 1,411 Mbp) showed that transcript discovery saturated when 99 to 132 million reads of a full set of 330 million PE reads were used. The addition of more reads increased the number of assembled transcripts, but these tended to be short and not informative [56]. Therefore, instead of sequencing deeper (i.e. more reads from the same sample), we recommend investing resources in either sequencing from more diverse samples (for example more tissues or more developmental stages) to generate a more comprehensive reference assembly, or more biological replicates for DGE analyses. Generally, the number of reads depends on the transcriptome complexity and can range between 2–5 million reads for insects with small genomes (e.g. *Drosophila melanogaster*) to 100 to 120 million reads for chelicerates with rather large genomes (e.g. *Parasteatoda tepidariorum*).

### Pre-processing and quality control of raw read data

Once a dataset of short reads in FASTQ format have been acquired several quality checks must be performed. While a variety of approaches exist for the targeted sequencing of different molecules (e.g. small RNAs, 16S and other PCR amplicons), we assume here that the desired short-read data represents mRNA transcripts, and therefore that the sequencing center has performed a polyadenylation selection step to reduce rRNA abundance during library preparation. As typically multiple samples are pooled for each Illumina run, all reads must be assigned back to their parent sample via the unique barcodes present in the sequencing adapters (i.e. de-multiplexing). The sequencing center will remove these barcodes, indices and other non-native sequences prior to providing them to the researcher in a FASTQ file. While in theory this means the researcher should be able to proceed directly to performing a de novo assembly, we strongly recommend assessing the raw data for read/base quality, read length, read number, GC composition and adapter contamination (see Table 1 for a set of tools that can perform these functions). Moreover, we strongly recommend to check for contamination either from unwanted organisms [57] or by rRNA [58, 59]. Contamination of mRNA-seq data by rRNA is often not investigated, however we have experience with samples from otherwise equivalently RNA-extracted replicates sequenced in the same run suffering from extremely high (85%) proportions of rRNA. Based on the results of these raw-read quality metrics, reads should be trimmed and low-quality reads should be removed (Table 1). It may take multiple iterations of trimming and filtering before a set of parameters is identified appropriate for the data being processed. Once the raw data has passed these quality controls, a de novo assembly can be performed.

**Table 1:** A list of software packages available for the various steps required to assemble a transcriptome de novo

de novo transcriptome assembly		
***Preprocessing and quality control of raw read data***		
FastQC	[60]	Read quality check
RNA-QC-chain	[61]	Sequencing quality and contamination trimming
RSeqQC	[62]	Read quality and distribution statistics
MultiQC	[63]	Summarization and visualization tool
HTQC	[64]	Read filtering, QC and visualizing
SortMeRNA	[58]	Filtering of rRNAs from meta-transcriptomic data
BBDuk (BBtools)	[59]	Read trimming, rRNA removal, filtering, error correction and much more
Trimmomatic	[65]	Read trimming, rRNA removal, filtering
***Assembly *de novo* single k-mer***		
Trinity	[66]	Each single k-mer assembler has its unique set of features and we encourage the user to systematically compare the outputs of different packages
SOAPdenov2	[67]	
IDBA-tran	[68]	
Trans-Abyss	[69]	
***Assembly *de novo* multiple k-mer***		
rnaSPADES	[70]	Each multiple k-mer assembler has its unique set of features and we encourage the user to systematically compare the outputs of different packages
SKESA	[71]	
Velvet	[72]	
***Transcriptome aggregation tools***		
TransPi	[73]	The principle of merging/aggregating the outputs of multiple de novo assemblies and then reducing redundancy lies at the core of these tools. The details of how they achieve this can generate divergent outputs which should be systematically compared
Cerveau and Jackson	[74]	
Nakasugi et al	[75]	
Mikado	[76]	
ConSemble	[77]	
***Quality assessment of *de novo* transcriptome assembly***		
Detonate	[78]	Model based score to evaluate transcriptome quality
TransRate	[79]	Quality assessment detecting chimeras, structural and sequencing errors
rnaQUAST	[80]	Based on reference genome and database
BUSCO	[81]	Based on single orthologue database
DOGMA	[82]	Based on protein domain database
Bellerophon	[83]	Result concatenation tool
***Functional annotation***		
Transdecoder	[84]	predicts CDS for each transcript
esl-translate	[85]	part of the HMMER package; reports all potential ORFs for each transcript
Trinotate	[86, 87]	Comprehensive functional annotation pipeline, generates easily accessible database with all annotation results
dammit	[88, 89]	Comprehensive functional annotation pipeline using reciprocal homology assignment
EnTAP	[90]	Integration of multiple functional annotations
***Differential gene expression analyses based on transcriptome references***		
***Read mapping to reference transcriptome***		
STAR	[91, 92]	Read mapper (traditional read alignment)
HISAT2	[93]	Read mapper (traditional read alignment)
Kallisto	[94]	Pseudo-aligner
Salmon	[95]	Quasi-mapper
RapMap	[96]	Quasi-mapper
***Transcript and mapping result grouping***		
Corset	[97]	Tools to reduce the diversity of reference transcripts inevitably generated from a de novo assembly – employed when performing read mapping for DGE
Grouper	[98]	
Compacta	[99]	
***Statistical analyses/differential gene expression***		
DEseq2	[100, 101]	Two of the most employed statistical tools for DGE analyses. They each employ different data normalization concepts and can therefore generate divergent results
edgeR	[102, 103]	

The issue of read normalization prior to performing a de novo assembly should also be mentioned at this stage. It has been reported that reducing the redundancy of reads originating from the same original transcript prior to assembly not only reduces the computational complexity of the problem (i.e. assembly time), but also the quality of the assembly can benefit from such measures [104]. Indeed, some packages include a read normalization step by default prior to any assembly action (for example Trinity; [105]) and different conceptional bases for read normalization have been used [104, 106]. As highlighted below for other parameters, we would encourage to empirically assess the effect of including/omitting read normalization on the final assembly (see below). Importantly, this normalization should not be confused with the mandatory normalization of data within and across samples during DGE analyses (see below).

### Assembly

Given the abundance of options, the novice (and expert) may have difficulty in deciding which assembly package to employ and which will give the “best” assembly. While there will rarely be a clear answer to this question (especially a priori), the QC steps that assess the quality of a de novo assembly (see below) will help guide the selection of the appropriate package. In general, we recommend using popular and highly cited short-read assemblers that have a respectable half-life in the literature and remain available and supported (Table 1) [107].

These short-read assemblers typically employ methods based on the construction of de Bruijn graphs which represent pathways of sequence-overlap through the raw data. Each graph represents a transcript or a group of similar transcripts [66]. During de Bruijn graph construction and the resulting assembly each read is divided into smaller fragments of size k, which must be shorter than the read length. These substrings of the reads, so called k-mers, are aligned against the collection of k-mers that exist in the raw data with an overlap of the length k-1. This iterative process therefore extends the aligned region one nucleotide at a time and continues until no additional overlap is found. Therefore, k-mer length has a strong influence on the final assembly and must be carefully chosen [108–110]. Excessively short k-mers will generate highly fragmented and duplicated assemblies but will detect rare transcripts, while long k-mers can miss rare transcripts but will resolve repetitive or error-prone regions. Transcriptome assemblers can be distinguished based on whether they employ a single k-mer value during assembly, or multiple k-mer values (Table 1).

A study based on 10 assemblers and 9 different datasets concluded that it is difficult to identify the ‘best assembler’ as their performance is intimately linked to the input data supplied [111]. The literature is replete with systematic comparisons of assemblers applied to the same input data that produce divergent outputs [68, 78, 112, 113], and our own experience reflects this. Therefore, we encourage researchers new to assembling transcriptome data to adopt a philosophy of performing multiple assemblies with a diversity of tools and settings, and systematically comparing their outputs (see below). In line with this approach, the ‘next generation’ of de novo transcriptome assemblers are aggregate tools that take the output from a variety of independent assembly packages, and either assess their individual completeness using a variety of metrics, or merge and then de-duplicate them according to similarity thresholds (Table 1).

### Quality assessment of de novo transcriptome assemblies

Evaluating the quality of a de novo assembled transcriptome is a key step that should precede its use in any downstream application. While the quality of an assembly can be best assessed based on prior genome or transcriptome data from the target organism (i.e. reference-based quality assessment), we focus our attention on reference-free tools because we are primarily concerned with scenarios focused on emerging model organisms. One of the first metrics of an assembly that should receive attention is the total number of transcripts (also often referred to as contigs). This value will be related to the total number of genes present in the genome of the organism and can therefore be predicted to be constrained to a reasonable minimum. For instance, metazoan models with well annotated genomes contain 10-22 k protein coding genes [114–116]. However, this guideline cannot account for mechanisms such as gene duplication and the presence of splice variants which will significantly inflate these numbers. Therefore, in addition to the number of transcripts, their length distribution should be considered as any transcriptome assembly will invariably include spurious short contigs that should be excluded from further analyses. In some reports a majority of transcripts are short [117] and can be removed without affecting downstream analyses. While it would be unwise to recommend a concrete threshold, many researchers ignore transcripts with lengths shorter than 200 bp (default threshold minimum transcript size for Trinity) and even up to 400 bp [118]. Any length threshold should be carefully chosen, for instance by taking the original read length and insert size (i.e. the distance between PE reads) into account. Generally, contigs shorter than the average read length should be excluded from the reference. It is important to note that frequently employed contiguity metrics, such as the N50 metric, which were developed for assessing the contiguity of genome assemblies [119] are uninformative in the context of transcriptomes because transcripts vary greatly in their lengths.

Another important measure of assembly quality is the average and/or total number of reads mapping to a contig as this metric allows identifying transcripts which are poorly supported by the original RNA-seq reads [80, 120]. By mapping reads originally used for the assembly back to the assembled transcriptome one can evaluate the proportion of reads used to generate the assembly and high-confidence transcripts supported by correctly paired reads can be identified and retained [121]. In addition, the distribution of reads across transcripts can be used to detect chimeras and other assembly artefacts [122]. Tools such as Detonate and TransRate (Table 1) can be used to map reads and generate a score that reflects the overall quality of the transcriptome by detecting chimeras, assembly, and sequencing errors. The ExN50 metric [123] represents a combination of transcript abundance estimation and transcript length to assess and compare the quality of transcriptome assemblies.

Another informative, and highly cited tool to assess assembly quality is BUSCO (benchmarking universal single-copy orthologs) [81]. BUSCO compares a transcriptome (or genome) against a curated database of single-copy orthologous genes. The concept is that if 90% of the BUSCO genes are in an assembly, then that assembly by extension is likely to be 90% complete for all genes. The tool provides various databases on different phylogenetic levels and the user selects the most appropriate lineage, such as all eukaryotes, plants, bacteria, or specific groups, such as spiders or molluscs. A BUSCO output will therefore return a meaningful estimate of the completeness, duplication, fragmentation, and lack of transcripts of genes that should be present in the assembly. Similarly, DOGMA assesses the completeness of a transcriptome by surveying the assembly for conserved protein domains [82] rather than complete genes. 

With the growing number of tools and approaches available to assess the quality of transcriptome assemblies, aggregate packages such as Bellerophon [83] have been developed and aim to incorporate multiple lines of evidence to identify an optimal assembly. In general, we recommend to systematically apply the outlined quality checks for multiple assemblies to facilitate relative comparisons.

### Functional annotation of a de novo transcriptome assembly

Once an optimal de novo assembly has been generated the next step is to predict putative functions of the proteins coded by the assembled transcripts. Such a functional annotation is commonly achieved by identifying similar (ideally homologous) sequences in protein or nucleotide databases. Any functional information assigned to the sequence in that database can be inferred to apply to the transcript in the de novo transcriptome assembly.

Typical protein or nucleotide databases are the UniProt (many proteins, non-curated)/SwissProt (fewer proteins, highly curated) [124] protein databases, as well as the most comprehensive, but non-curated NCBI databases for protein (NR) and nucleotide (NT) sequences [125]. The search for homologous sequences in such databases is often a time-consuming computational task because it mostly relies on tools employing the Basic local alignment search tool (BLAST) logic [126]. As protein sequences tend to be more conserved than nucleotide sequences, it is generally computationally less demanding to perform the homology searches in protein databases using translated sequences as input. Therefore, a typical first step of a functional annotation pipeline is to identify the coding sequence (CDS) and/or the longest open reading frames (ORFs) for each transcript in the de novo assembly (Table 1). Once sequence homologs are identified, the functional information associated to them is assessed employing functional databases. For instance, the InterPro database contains information about protein domains and families [127] and the gene ontology (GO) knowledgebase is a highly curated collection of functional information (e.g. biological function, molecular functions) for genes and gene products derived predominantly from model organisms [128]. The Kyoto Encyclopedia of Genes and Genomes (KEGG) database allows placing de novo transcripts into the context of biological networks and pathways [129, 130]. To streamline the major computational steps required for a functional transcriptome annotation, comprehensive pipelines, such as Trinotate and dammit (Table 1) have been established. The output of such pipelines are easily accessible files, such as Excel spreadsheets or a Sqlite database, as well as more advanced annotation files for further bioinformatic analyses. Potential problems during functional annotation caused by the typically high number of transcripts are for instance addressed by the Eukaryotic Non-Model Transcriptome Annotation Pipeline (EnTAP) (Table 1), which filters transcripts based on expression levels and coding potential and subsequently combines multiple functional annotations (i.e. based on annotation pipelines) into one high-confidence annotation.

The quality of the functional annotation strongly depends on the ability to identify clear homologous sequences, as well as on the quality of the functional information stored in these databases. If the de novo transcriptome is derived from an organism that is related to a model organism, sequence homology can often be easily established, and putative functions can be assigned for many transcripts. However, if relatives of the study organism are not well represented in typical databases, it may be challenging to functionally annotate the majority of transcripts. Hence, depending on the phylogenetic relation to organisms for which high-quality functional information is available in databases, the functional annotation should follow slightly different routes. For instance, while BLAST searches in highly curated protein databases are most time- and resources-efficient, it may be advantageous to base the homology assignment on larger protein databases or even more comprehensive nucleotide databases if many transcripts remain un-annotated after a first annotation round. It is also important to note that it may not be possible to assign putative functions to lineage-restricted or novel, as well as fast-evolving genes in the de novo assembly as they tend to lack homologous sequences in the respective databases. In these cases, putative functions may only be deduced from the annotation of short protein domains rather than comprehensive functional information. A final consideration is related to the purpose of the de novo assembly. If the assembly is intended to provide a broad overview of the transcripts expressed in an organism, a full functional annotation is desirable. However, if the major goal is to identify expression differences between conditions, it could be sufficient to functionally annotate only those transcripts that show significant differences in gene expression. It must be kept in mind that various scenarios of protein evolution, for example domain shuffling within and between proteins, may complicate the interpretation of superficial annotation efforts.

### DGE analyses based on de novo transcriptome assemblies

To establish causal relationships between the genome and biological phenomena, gene expression studies most of the time represent an easy entry point to identify lists of candidate genes that could be responsible for a certain phenotypic outcome. The analysis of differential gene expression from RNA-seq data is therefore one of the most employed methods in molecular biology nowadays, and many best-practice guidelines are available [21, 22, 131–134]. Here, we briefly summarize the major analysis steps, standard recommendations and we emphasize special requirements when de novo assemblies are used as the reference.

### Experimental design, sequencing and pre-processing of RNA-seq reads

Careful planning is a critical phase of any DGE experiment and will save significant time and financial costs in the later phases of a project. The most common first decision relates to the number of replicates to generate. A study specifically aimed at answering this question in the well-established unicellular yeast model system S*accharomyces cerevisiae* found that 20 high quality replicates in a simple 2-way comparison were required to detect > 85% of the significantly differentially expressed genes (regardless of fold-change). With a commonly employed experimental design of 3 replicates only 20–40% of all significantly differentially expressed genes could be detected [135]. From such studies a general recommendation of 6 biological replicates for all experimental conditions emerged, while 12 replicates are recommended when the identification of the majority of differentially expressed genes is required [135].

Beyond replication, factors related to the Illumina sequencing platform and the generated reads should be considered at the project planning stage. A general consensus emerges from the literature that recommends stranded sequencing in order to maximize the disambiguation of potential read placement (i.e. caused by overlapping gene bodies on different strands) [136], read lengths of 50–100 bp [137] and 25–100 million reads per sample/replicate (depending on the genome/transcriptome size) [138]. Unless splice-variant quantification is paramount [137], SE reads suffer no disadvantage to PE reads [139]. On the contrary, SE reads usually provide more sequencing depth for the same cost and they often generate higher mapping rates. However, improvements of the library preparation and sequencing protocols, diminishes the cost differences between PE and SE reads. A dedicated study showed that 40 bp PE reads resulted in more consistent gene expression estimates compared to 75 bp SE reads [140], while the same costs apply for both approaches. We consider the number of biological replicates, followed by sequencing depth, to be the two primary variables that should be considered in any DGE experiment, and we strongly recommend discussing these main parameters closely with the sequencing center.

Raw short reads provided by the sequencing center should be subjected to proper quality assessment, trimming and filtering as described above to obtain high-quality short reads to proceed with (Fig. 3). Specifically, the identification of samples with high percentage of rRNA reads will avoid artefacts associated with inaccurate estimates of library sequencing depth and therefore transcript coverage during DGE analysis [141–143].

In case the same RNA-seq reads will be used for de novo assemblies and subsequent DGE analyses (Figs. 1B and 2C), it is important to note that the read normalization step that may be done prior to the assembly must be omitted prior to the DGE analysis as this step will eliminate all differences in expression. Moreover, proper replication is required, even though the assembly per se does not require replicates. The type of sequencing as recommended above (Fig. 1B) represents an acceptable compromise between short SE reads for quantification and long PE reads for the assembly.

### Read mapping, transcript quantification and statistical analyses

Mapping RNA-seq reads to a reference (whether a de novo transcriptome assembly or an annotated genome) lies at the core of any DGE analysis. A multitude of freely available read-mapping tools now exist (Table 1 for a selection) and the mapping quality can be software dependent [144]. Accordingly, there is no shortage of studies that compare the performances of different tools [48, 49, 145–147]. More traditional read-mapping algorithms (e.g. STAR and HISAT2, Table 1) are splice-aware, and have been reported to be more accurate with regards to miRNAs and lowly expressed genes [148] than pseudo-aligners (e.g. Kallisto, Table 1) and quasi-mappers (e.g. Salmon and RapMap, Table 1). Conversely, these more recent read-mapping tools have been reported to be more accurate than splice-aware methods for the quantification of long non-coding RNAs [149]. Overall, we advise the reader to appraise the strengths and weaknesses of the selected mapping tool given the primary goal of the read-mapping exercise, such as mRNA quantification, miRNA quantification or isoform quantification.

One issue that all read-mapping tools must deal with is how reads that mapped with equal fit to more than one location in the reference are handled. Such ambiguous read-mapping rates can be as high as 37% [150] and they could for instance be the result of splice-variants, duplicated genes, pseudo-genes and low complexity genes in the reference. This issue is particularly important when de novo assemblies serve as the mapping reference because each gene is often represented by many transcripts (whether true isoforms or spurious contigs). Several strategies exist to deal with multi-mapping reads including eliminating these reads from the DGE analysis, splitting them equally between their possible true origins, assigning them to a location based on a probability distribution constructed from unambiguously mapped reads, or collapsing the potential targets into gene groups and providing expression level estimates for these groups rather than the individual genes. While there appears to be no consensus on this issue [reviewed in 109], and new solutions are being actively developed [151, 152] we recommend that any DGE analysis should compare the impact of ignoring multi-mapping reads (i.e. only considering uniquely mapping reads) vs. assigning them to a location using at least one explicit strategy. If a de novo transcriptome assembly serves as the mapping reference, multiple tools have been established which cluster transcripts based on the likelihood of having the same reads mapped to it (e.g. Corset, Grouper, Compacta; Table 1). This allows gene expression to be estimated on the level of clusters, rather than individual transcripts. Another option to reduce the complexity of the transcriptome reference is to only keep the longest isoform for each putative gene locus. This approach will only result in reliable results if the isoform-clustering of transcripts is performed correctly. 

Importantly, some RNA-seq methods rely on sequencing only the 3-prime ends of RNA fragments (e.g. QuantSeq, [153]) or focus on transient RNA molecules (e.g. TT-seq, [154]) and they profit from or require a well-assembled and annotated reference genome to unambiguously assign RNA-seq reads to specific genes [155, 156]. Also commonly employed droplet-based single-cell RNA-seq technologies (such as the 10X Genomics platform) rely on 3’ end sequencing [157], and 25% of the reads generated in single-nucleus RNA-seq data typically represent intronic sequences [158]. Therefore, a reference genome is often a prerequisite when gene expression is being assessed at single-cell/single-nucleus resolution. These special expression quantification methods are therefore not advisable if only de novo assembled transcriptome references are available.

After read mapping, a count matrix summarizing the number of reads mapped to each gene/transcript in each biological condition and replicate is generated and used as the input for the statistical assessment of expression differences. In summary, gene expression data is typically modelled based on a negative binomial distribution to estimate the mean gene/transcript expression, as well as the variance among replicates and to normalize the read counts for differences in the library size (i.e. total number of mapped reads) (e.g. DEseq2, edgeR; Table 1). A statistical comparison of the mean expression between conditions results in an adjusted p-value (after accounting for multiple testing), which can be used to identify a list of significantly differentially expressed genes (i.e. genes whose adjusted p-value falls below 0.05 or 0.01 for more conservative estimates). It has been demonstrated that the mainstream DGE software tools differ in their abilities to identify differentially expressed genes and in their rates of false positives [159]. To minimize this source of technical bias, tools have been developed by the community to take the consensus of multiple DGE analysis outputs and this has been shown to increase the robustness of DGE predictions [160]. Moreover, filtering out genes/transcripts supported by low read counts (for example less than 10 reads) is a common practice in RNA-seq data analysis, and can increase the number of differentially expressed genes detected and improve the sensitivity of DGE analyses [161, 162]. 

It is important to distinguish between the two major applications of transcript abundance estimation. The procedure outlined above assumes that raw read counts for each transcript are compared between biological/experimental conditions. Hence, the read counts to be compared are mapped against the same reference transcript/gene and accordingly the transcript length does not need to be considered. In contrast, comparisons of expression levels of different transcripts/genes within the same sample are regularly employed to generate heatmaps and principal component analyses (PCA) plots to globally describe the expression data. Moreover, abundance estimates across transcripts are typically used during the quality assessment of de novo assemblies to identify low-quality transcripts (see above). Statistical tools used for such cross-sample comparisons should not be used in this case as they do not account for differences in transcript/gene length. Instead, normalization procedures which account for library size and transcript/gene length such as RPKM (reads per kilobase of exon per million reads mapped) and its derivates FPKM (fragments per kilobase of exon per million fragments mapped) and TPM (transcripts per kilobase million) should be employed [163–165]. Special caution must be also applied if gene expression between different populations/species with different mapping references should be compared. A study in different *Drosophila* species showed that despite library size/transcript length normalization prior to the DGE analysis, false-positive significantly differentially expressed genes were identified [33]. To our knowledge, the problem of interspecific expression comparison is not yet conclusively solved.

## Conclusion and outlook

Generating a de novo transcriptome for an emerging model organism represents a significant challenge for beginners, with many different scientific and technical aspects to consider. Data quality benefits from a clear communication with the sequencing center generating the raw data. Moreover, the quality and selection of open-source software tools that exist today, along with an extensive scientific literature and advice from colleagues can greatly support the journey from the initial scientific question to the discovery of the genes associated with a trait of interest. While comprehensive de novo assembled transcriptomes are excellent starting points to study and compare the gene content of an organism, DGE studies promise exciting new insights for subsets of biological processes or tissues. However, without tempering the excitement, it is important to keep in mind that any DGE analysis typically results in long lists of differentially expressed genes. These initial lists should always be regarded as candidates for further investigation, rather than the final answer to a question. Often the real work begins with these lists and will require a range of additional computational analyses and wet lab experiments that should verify and test any resulting interpretations. While beyond the scope of this review, computational approaches to narrow down these often daunting lists of candidate genes may include assigning GO terms [166–168], or pathway enrichment analyses [169] which allow identifying specific sets of genes or perhaps an entire signaling pathway that is significantly up-regulated in the tissue/condition of interest. As these enrichment analyses are based on homology assignments, the downstream analysis pipeline should be flexible enough to accommodate unexpected outcomes, such as novel, fast-evolving, or lineage-restricted genes for which no functional data may be available in existing databases. Following the rational that genes with similar expression profiles are likely to be co-regulated, one can also reconstruct co-expression networks [170], which place individual candidate genes into a systemic regulatory context. Such networks may be very powerful to link uncharacterized candidate genes to genes with known functions. Eventually, independent verification of any DGE or network analysis should be performed. This can be achieved quantitatively (for example via qPCR), spatially (e.g. in situ hybridization) or functionally using gain- and loss-of-function methods (RNAi or CRISPR). The selection of candidate genes for such further studies could be based on their homology with genes known to be associated with similar traits in other model systems.

As new sequencing technologies are continuously and rapidly being deployed it is difficult to predict how the analysis of genomes and transcriptomes will evolve. What is certain is the identification of genes associated with biological traits of interest will continue to fascinate scientists from a variety of disciplines, from medicine to evolution to agriculture. By using the methods and techniques we have surveyed here, these questions can be readily addressed in organisms that do not have the historical pedigree that traditional models enjoy.

## Data Availability

Not applicable.

## Abbreviations

bp: Base pairs
BLAST: Basic local alignment search tool
BUSCO: Benchmarking universal single-copy orthologs
cDNA: Complementary DNA
DGE: Differential gene expression
mRNA: Messenger RNA
PE: Paired-end
QC: Quality control
qPCR: Quantitative polymerase chain reaction
RNAi: RNA interference
rRNA: Ribosomal RNA
SE: Single-end
